# Professional Self-Positioning of Indian Social Workers in Response to Domestic Violence

**DOI:** 10.1177/0886260520922920

**Published:** 2020-05-21

**Authors:** Sisko Piippo, Pasi Hirvonen, Janet Carter Anand

**Affiliations:** 1 University of Eastern Finland, Kuopio, Finland

**Keywords:** domestic violence, domestic violence interventions, battered women, India, positioning theory, social workers

## Abstract

This article explores the self-positioning of Indian social workers who work with female survivors of domestic violence (DV). We know from previous research about the experiences of persons who have encountered DV, but more studies on the point of view of the professionals is needed. Relying on positioning theory and discursive analysis as a framework, we analyzed interviews (*N* = 18) concerning the practices, emotions, and attitudes of social workers. Positioning theory enables scholars to approach the situation of encountering a survivor as a social event, which consists of the meaning-making activities of social workers. Professionals self-position themselves as one or a combination of the following: (a) a challenger of gendered oppression, (b) an advocator of women’s rights, (c) a facilitator of women’s empowerment, and/or (d) a self-reflector of personal emotions and attitudes. The findings suggest that the event of DV intervention is a discursive and contextually situated moral practice in which the distribution of rights and duties to say and do things is of particular interest. The act of self-positioning is constructed in relation to sociocultural settings. Social workers may take several positions during the meetings with survivors. Positions build our understanding of how social workers in violence-specific units make sense of DV, illustrating how the act of self-positioning may also define to the position of the survivor. Social workers experienced boundaries, and possibilities in responding to DV were explored. The study reveals that barriers should be addressed at a wider level by funders and policy makers to enhance the continuity of work and the implementation of women rights legislation in India.

## Introduction

This article addresses the insights that are gleaned from Indian social workers who work with female survivors of domestic violence (DV), emphasizing workers’ views on how they position themselves as professionals when responding to cases of DV. By analyzing discourses focused on work-related practices, emotions, and attitudes, the article examines the perceived rights and duties of social workers to intervene DV. In this article, we approach violence in Indian family structure, where several generations live together as a joint family. Thus, we use the concept of DV, which refers to the violence occurring among household members. India is an example of a powerful nation with relatively fast economic growth. However, concerns about human rights have become more visible globally in the 21st century. As an example, India has been criticized for its low level of commitment to international human rights treaties and for failing to protect women from DV (Human Rights Watch, 2017; Kim, 2018).

As authors, we are aware of the legacy of colonialism and resistance, which can be awakened by the oversimplified idea of “transplanting” Western principles and ideology to “empower” women in the Global South (Rajan, 2018). Also, we recognize the importance of avoiding talking about Indian women as a homogeneous, oppressed group (Mohanty, 1995). At the same time, we conclude that at the state level, there exists no treaty-driven human rights system to protect women in India and to enhance women’s position to exercise their rights (Kim, 2018; Tatiya & Vidyasagar, 2010).

Social change, empowerment, and the liberation of people in the name of social justice and human rights are cited as central aspects of social work (International Federation of Social Work [IFSW], 2014). Considering DV as a social problem and a human rights violation (Garcia-Moreno et al., 2005), the professional responsibility of social workers to intervene becomes evident. However, DV—and intervening in DV—may cause moral dilemmas for professionals in relation to their rights and duties (e.g., Kethineni et al., 2016; Pecnik & Bezensek-Lalik, 2011; Virkki & Jäppinen, 2017). Previous research has shown that the demands of dealing with survivors in sensitive and self-reflexive ways (Kulkarni et al., 2012; Lapierre & Côté, 2011) conflict strongly with findings that report survivors’ experiences of dissatisfaction with the service system in the Global South and North alike. The responsibility of the perpetrators for their violent behavior may be ignored, and the survivors themselves held responsible for the violence (e.g., Keeling & Van Wormer, 2012; Kim, 2018; Nikupeteri, 2017).

In addition to its physical form, DV may exist as emotional, sexual, financial, psychological, and social control, and, more generally, as any form of controlling behavior (Garcia-Moreno et al., 2005). Although DV has been observed globally, it is also expressed differently in specific cultural contexts. In India, women can be exposed to dowry-related violence and honor-based violence, which are usually perpetrated by her husband or in-laws (Menon & Allen, 2018). Gender inequality in India is complex and intersectional in nature, and it should be understood in the context of traditional norms of gender, poverty, and religion (e.g., Rao, 2013). Together with a lack of education and awareness, these are approached as major obstacles in popularizing and internalizing women’s rights (Nadkarni & Sinha, 2016). The Gender Inequality Index ranks India 125th with regard to reproductive health, empowerment, and labor market participation (Human Development Report, 2016). According to the National Health and Welfare Study, 31% of Indian women aged 15 to 49 have experienced spousal violence (International Institute for Population Sciences [IIPS] and ICF, 2017). The literature suggests that this is underreported due to a neglect of women’s rights: only a minority of abused women seek help, due to the high societal tolerance of DV, including a victim-blaming mindset and the social stigma associated with divorce or separation (Barik, 2011; Kim, 2018; Nadda et al., 2018).

In 2005, the Protection of Women from Domestic Violence Act was passed, demonstrating a step toward committing to change. However, addressing DV remains challenging (Kim, 2018). As Ahmed-Ghosh (2004) states, a patriarchy highlighting traditional family values is well explicated in government statements and legal systems. In India, the social hierarchy in a joint family can make a woman vulnerable to violence perpetrated not only by her husband but also by her in-laws and relatives. Despite nationally reported difficulties with implementing the law (Kim, 2018; Nadda et al., 2018), initiatives enhancing women’s rights have been developed (Kulkarni et al., 2012; Pandya, 2014). Kethineni et al. (2016) noted that the lack of a formal system for protecting women has triggered women’s organizations to take a leading role in DV services. Women’s rights activists have had an active role in highlighting violence against women in the national debate (Pandya, 2014).

## Social Work and the Cultural Context of DV in India

The self-positioning of Indian social workers in response to DV cannot be understood without reference to the political, social, and occupational contexts of exercising practice. Social work involving charity and community work with a spiritual and ethical ideology started long before formal social work was institutionalized in the 1930s (Nadkarni & Sinha, 2016; Ranta-Tyrkkö, 2010). The development of professional social work has been described as complex and has been criticized for being colonized by Western theory and dismissing indigenous features (e.g., Dash, 2017; Ranta-Tyrkkö, 2010). As Rao (2013) stated, Indian social work, as well as work with survivors, cannot be viewed via the concept of Western individualism. During the past few decades, there have been significant efforts to develop Indian-based curricula and integrate country-related phenomena into social work education (Dash, 2017). Indigenous thinking and doing has actively refined and even challenged Western theories and approaches. However, violence against women has not become established content in social work curricula (Barik, 2011). Adopting a women’s rights approach may require social workers to take an opposite position to governmental policies, which may be considered challenging if organizations are dependent on government funding (Nadkarni & Sinha, 2016).

Social work practice in India in the field of DV differs from the roles and functions of practitioners in European countries for several reasons. Unlike in many Western countries, social work is not a registered profession in India. The state is often criticized for failing to regulate schools of social work in terms of curricular development (Nadkarni & Sinha, 2016). Also, much work is required to legitimize social work as a sanctioned service, as nongovernmental organizations (NGOs) employ most social workers (Chakraborty et al., 2017; Dash, 2017; Rao, 2013). Social work, with its low professional status, lacks social recognition. For example, a social work degree is required only in a few places, and wages are low (Dash, 2017; Rao, 2013).In NGOs, work is tied to external funding, and social workers may be expected to participate in fund-raising activities. Therefore, social work requires dedication, commitment, and, to some extent, a social and political calling. Volunteers who undertake social work roles have traditionally made a significant contribution to DV work, especially in rural settings. A person with a social work education can also work as a volunteer, organizing DV services such as self-help groups (e.g., Chakraborty et al., 2017).

The topics of the experiences, emotions, and attitudes of Indian social workers and their relation to DV are relatively unexplored. Husso et al. (2012) suggested that from the professional’s standpoint, the dilemma of how and when to intervene in DV cases appears to be a question of meaning and sense-making regarding the professional’s role and responsibilities. Problems related to DV rarely present themselves as a given. Instead, they are socially constructed and need to be formalized by sense-making (Husso et al., 2012). By utilizing positioning theory (Harré & Van Langenhove, 1999), our study approaches this sense-making process by focusing on the following question: How do Indian social workers position themselves as they make sense of their role and their rights and duties when working with survivors of DV? Only a few studies in social work research have applied positioning theory when researching sensitive topics (see Jarnkvist & Brännström, 2016; Nikupeteri, 2017), and none have explored the self-positioning of Indian social workers in the context of DV.

## Positioning Theory as a Framework

Stemming from social constructionism, positioning theory regards positioning as a discursive and contextually situated moral practice in which the distribution of rights and duties to say and do things is of particular interest (Davies & Harré, 1990; Harré, 2012; Harré & Van Langenhove, 1999). Negotiations and meanings related to those rights and duties may become relevant—whether intentionally or unintentionally—in everyday interactions (*interactive positioning*) or self-reports, when someone positions themselves (*reflexive positioning*) (Davies & Harré, 1990). By identifying the reflexive self-positioning of social workers through interview transcripts, this article explores the rights and duties of social workers when they encounter survivors. Through its focus on moral order—the basis of context-specific appropriate behaviors (Harré & Van Langenhove, 1999; Van Langenhove, 2017)—positioning theory offers an applicable framework for investigating sensitive topics with several ethical tensions.

Regarding positioning, three mutually determining elements of social and professional behavior are considered in this study. First are the *positioning acts* of the professional in any given social episode of interaction. Second are the actual *positions* that professionals occupy in relation to assigned rights and duties and adopted in these positioning acts. Third are the *story lines* that unfold as a consequence of professional interaction (e.g., Harré, 2008, 2012). Each story line unfolds in relation to the local moral order originating from different discursive practices. Van Langenhove (2017) outlined the varieties of moral orders as legal, cultural, institutional, conversational, and intrapersonal. For example, the story line of a professional social worker might be constructed in relation to institutional (governmental and nongovernmental) and legal (authorized position) moral orders.

Articulating that the individual should be explored in relation to one’s social and cultural context, positioning theory is applicable to international research on social work. Problems and solutions are no longer country-specific, although their outlooks and consequences vary from place to place (Ranta-Tyrkkö, 2010). Consequently, we assume that by analyzing the practices, emotions, and attitudes toward DV work as discussed by Indian social workers, it will be possible not only to provide valuable country-specific information on their rights and duties but also to highlight the global nature of their work with survivors.

## Method

### Participants

The first author collected data from two focus group interviews and four personal interviews (*N* = 18) during a 3-month exchange period in 2018 at an Indian university. With the help of local scholars, she recruited participants from local NGOs who specialized in working with DV survivors.

First, two focus group interviews were conducted (Table 1). As a method, a focus group interview gathers a homogeneous group of persons together who are assumed to have the best information on a specific area. It also offers the possibility of exploring different opinions and attitudes arising from discussions (Fern, 2001; Silverman, 2014). The first group had six participants, and the second eight. Interviews, facilitated by the interviewer, were conducted in participants’ workplaces. Discussions were vivid and the ideal of guaranteeing equal participation (Brinkmann & Kvale, 2015) was valued and supported. Language produced some challenges; some participants spoke Marathi, and members of the group translated their speech into English. An external interpreter would have enhanced equality of participants. Individual interviews with four social workers were conducted to deepen the data. One participant represented an NGO from an earlier focus group, but three social workers worked in other NGOs.

**Table 1.** Social Workers’ Interviews (*N* = 18).

Background information	Focus Group 1 February 2018	Focus Group 2 March 2018	Individual Interviews March–April 2018
Participants	6 (5 female, 1 male)	8 (all female)	4 (all female)
Duration of interview	1 hr 22 min	1 hr 52 min	59 min (average)
Educational background	6 MSW	5 MSW, 3 BA and BE	4 MSW
Working experience with survivors (average)	2.2 years	11.7 years	7.3 years

All interviews were semi-structured and thematized (Silverman, 2014), as follows: *encountering violence*, *multiprofessional work*, *education*, and *future visions.* For example, forms of DV, work with survivors, work with perpetrators, and the feelings that the work aroused were all discussed. In recruiting participants, researchers believed that a congruent educational background would promote data homogeneity. Fifteen interviewees had Master of Social Work degrees. Three participants had Bachelor of Arts or Bachelor of Education degrees and had completed authorized courses in counseling. Considering their similar job descriptions as social workers, researchers included all participants in the analysis. In the quoted text that follows, G means groups, P the participant of a group, and I to an individual interviewee. All data excerpts are from female participants.

### Analysis

The transcripts were read and reread in ATLAS.ti software to find extracts in which social workers described their work. First, we utilized thematic analysis (Silverman, 2014). Special attention was paid to sentences in which practices, emotions, and attitudes were described. Themes were categorized and inductively combined into groups. However, we recognized features of overlapping occurrences and the controversial content of some essential themes. That led us to consider the possibility of applying positioning theory to track the wider dimensions of the phenomenon and to approach tensions emerging from cultural factors that shaped an understanding of DV, as outlined earlier.

Second, four main themes to be used as an analytical tool (Nikupeteri, 2017) were constructed: power, rights, agency, and self-understanding (Table 2). We continued by exploring how positioning acts were expressed in relation to these themes, whether intentionally or unintentionally, through participants’ speech as elements of social episodes (Harré, 2012). We utilized analytical concepts such as self–other, but through positioning theory, we could focus on the intrapersonal factors of self-positioning and reflexive positioning (Davies & Harré, 1990) and not on face-to-face interactions between interviewees.

**Table 2.** Social Workers’ Positions.

Position	Theme	Story line	Nature of Practice
Challenger of gendered oppression	Power	Story line of reflexivity	Political
Advocator of women’s rights	Women’s rights	Story line of education	Juridical
Facilitator of personal empowerment	Women’s agency	Story line of counseling	Psychosocial
Self-reflector of personal emotions and attitudes	Self-understanding	Story line of reflectivity	Intrapersonal

Third, after identifying four positions, the analysis progressed with the story lines by identifying the rights and duties of social workers and the nature of social work practice. The development of social episodes follows story lines—preestablished patterns—that are also cited as loose clusters of narrative conventions (Table 2). A conversational history of what has already been said about an issue in the form of previous research was utilized to deepen the analysis (Harré, 2012).

### Ethical Considerations

Ethical guidelines of the University of Eastern Finland and Finnish Advisory Board of on Research Integrity (2012) were complied with. The interviewer discussed the research with the managers of NGOs, who recruited volunteer participants. In addition to receiving collective consent from the managers, the researchers received informed, oral consent from the participants after they had been told the aims of the research, how the information would be used, and how the data would be stored (Brinkmann & Kvale, 2015). Voluntary participation was emphasized. Participants were aware that they could withdraw their consent at any stage without consequences. Full anonymity was guaranteed.

The authors were aware of cultural tensions that can be caused when Western scholars conduct research in the Global South. To relieve such tensions (Thompson, 2011), special attention was paid to respecting participants and building confidence (Silverman, 2014), for example, by explaining the duration of the interviewer’s stay (3 months) and her efforts to learn the Hindi language as well as by contacting participants after the interviews and asking participating social workers to comment on the draft. For disseminating purposes, the first author presented preliminary findings in a symposium and in a conference in India in autumn 2018.

## The Self-Positioning of Social Workers in Response to DV

The results of the study are summarized, using four differing positions that participant social workers occupied. They are illustrated with quotes and described with reference to the story line and response focus.

### Challenger of Gendered Oppression

While discussing gendered power with reference to the story line of reflexivity, the position of challenger of gendered oppression was adopted. Reflexive consciousness about social divisions and the power dynamics within broader social structures (Thompson, 2011) were illustrated, and DV was approached as a matter of social injustice needing to be tackled on the political level in discussions concerning the oppressive power structure as a cause of DV, especially within the family. In the first extract, the challenger position is of a tacit nature, as the social worker describes the dominant patriarchal power relations.

In India, it’s a patriarchy. We have a very strong patriarchy, which gives men a lot of power. (I4)

We listen to another woman, we feel that sometimes it’s very difficult … And we see them suffering, but we cannot do anything. They think that this is our life, we have to tolerate this … And we realize that the main cause of all this violence is gender discrimination. (I1)

The challenger position is constructed in a dynamic fashion between the self-positioning of the social worker and the other-positioning of the survivor, who was assumed to be in a powerless position. Explicit self-positioning (Harré & Van Langenhove, 1999) takes place in the second extract, because one of the participants positioned social workers collectively (“We listen,” “we feel”) as being obligated to take action but powerless to help when deeply rooted societal and cultural causes of DV were discussed.

Social workers reflected oppressive power relations in the family structure when discussing cases in which giving birth to a girl child led to abuse. Female abortions and female child murders were also discussed as being external examples of gender discrimination and male control over women’s lives and bodies. The social worker’s challenger position was constructed tacitly by describing and reflecting on the matrimonial institution and questioning the dowry tradition, taking this tradition as an example of how patriarchal norms reproduce an economic motivator for violence.

Even that kind of … arrangement is very exploitative. Because she goes to her husband. She doesn’t have a house in her name … It’s a very institutionalized mindset, very structured, the more money the victim brings in … and of course they want somebody to do domestic work in the house. Somebody who doesn’t talk, somebody who doesn’t question, somebody who follows the rules, somebody who listens to everything they say. (I2)

I remember one case, between [a] husband and wife there was no problem at all. It was the in-laws that tried to create problems between them so that they would be separated. And why? The girl did not bring enough of a dowry. It took us almost six months to understand the root causes … So, we could save the marriage but definitely we had to separate the in-laws from the husband and wife. (I3)

A bride is expected to move into the matrimonial house, where several generations of the groom’s relatives live together. As part of the marriage arrangements, the bride’s family is typically expected to give some property (cash, household items) to the groom’s family, despite the existence of the Dowry Prohibition Act since 1961. Demands for dowries for several daughters may cause economically disadvantaged parents unreasonable distress, leading them to undertake desperate action. Ultimately, a girl child is brought up for another household and lineage; she will not be there to secure her parents’ welfare in their old age (Ahmed-Ghosh, 2004). In addition, as expressed in the latter extract about the social worker’s reflexive self-positioning, dowries estimated as inadequate by in-laws may lead to demands for extra property and to a bride’s harassment, torture, and even murder if these demands are not met. Particularly with regard to collective positioning, the duty of social workers to challenge the oppressive family relations (“*we had* to separate”) behind DV became evident. Their right to intervene was not explicated as being recognized before the intervention. Instead, by using third-order positioning when reflecting on the case afterward (Harré & Van Langenhove, 1999), the worker implied the justification for the intervention in the positioning act: “W*e could* save the marriage.”

### Advocator of Women’s Rights

The position of social workers as advocators in relation to women’s rights follows the story line of education unfolding into the juridical nature of the work. The positioning acts not only referred to survivors but also to men and network institutions, illustrating the psychoeducational aspect of work with survivors.

Because these women don’t know what the abuser did. That’s why it’s the role of social workers to understand … the abuse. (G1P1)

We try to make her understand her own rights, financial or mental. We try to make her understand and make her stronger to make her own decisions. To make her fight against abuse. (G1P4)

The social worker’s position as an advocator is constructed through explicit collective positioning by emphasizing the duty of social workers to promote survivors’ self-awareness. Women’s acceptance of violence evolves from both cultural and legal norms. For example, marital rape is not an offense, and some religious laws prescribe having sex with one’s husband as a wife’s duty (Barik, 2011; Kim, 2018). Thus, work in the field of sexual violence requires time and special sensitivity. Due to male dominance in society, participants emphasized their position in relation to men as they advocated for women’s rights. The participants agreed that preventive work with boys and young men is significant for nurturing attitudinal change in future generations. However, tensions exist when encountering perpetrators.

We work with men because women are dependent on men. So, in entitling her to her rights, we have to work with men. (G1P3)

They always deny. It was her fault, she provokes me. She doesn’t treat the children properly, she talks with men, she came home late from work, she doesn’t do her housework, she complains about my mother. These same stories you hear from every abuser … They said that she burned herself, I didn’t do it, she’s lying. So actually, we call the abuser only to tell them “Hello, we are here now.” That’s the only purpose. To tell him that we are watching. It’s like a warning, a preventive strategy for further violence … We know, I know, that there’s no role I can play with abusers. (I2)

An advocator position is constructed through explicit collective positioning (“*We* work”). Highlighting the complex nature of the work, the position concerns not only the social worker’s right to advocate but also their duty to hear both parties. This obligation was implemented in different ways, emphasizing the survivor’s safety and her willingness to cooperate as priorities. Some participants emphasized the importance of having regular meetings and a nonjudgmental mentality with perpetrators. However, the purpose of contacting the perpetrators can be simply to make them aware of the social worker’s presence. This also illustrates challenges when undertaking their duties: To encounter abusers brought up in a world of male dominance who express an unwillingness to admit to violent behavior. Interestingly, at the end of the latter extract, when the social worker ultimately dissociated herself from encountering the abusers, a shift in collective positioning from “we” to “I” occurs, illustrating an awareness of divergent views.

Discourses concerning interprofessional cooperation illustrate structural advocacy for women’s rights. That highlights the expert position and the social worker’s experienced right to educate and instruct.

We go to shelter homes, we go to other NGOs, we go to protection offices, we go to police stations, we go to the court to help the woman. Helping entitlement of their rights. (G1P2)

In hospitals, social workers had an active role in preventive work when training nurses and doctors to launch routine DV screenings. Social workers from an NGO discussed how they gave a presentation to a parliamentary committee in the planning stage of the Domestic Violence Act, 2005. They also described how various training programs were organized by NGOs to tackle network partners’ negative attitudes, such as views of DV work as “extra work” and a lack of knowledge regarding DV legislation. Raising the awareness of women’s rights among the police was essential, because registration of DV cases is a prerequisite for survivors to receive further services such as free legal aid.

But the police don’t respond properly. They don’t write the reports needed. They don’t write. They send them back. They say “Ok, this is your family matter, [unclear] you manage yourself, don’t come to us.” (I1)

However, intervening in a private matter such as DV was critically defined as a task “beneath their [police] dignity” (I4). The previous extract demonstrates how social workers not only depend on infrastructure to support rights, but also a justification of the duty of social workers to advocate for women’s rights with the police.

### Facilitator of Personal Empowerment

Social workers’ position as facilitators was constructed in relation to a woman’s agency. Following the story line of counseling, social work practice appears as psychosocial. Descriptions regarding client independence are illustrated in the next extract, whereas the two subsequent extracts discuss themes relating to individual decision-making.

In discussions among social workers, women were often described as being dependent upon men in Indian patriarchal society. The duty of social workers was defined as supporting survivors in their move toward independence.

We try to provide information. We do that, so that women would be able to make decisions for themselves. Sometimes we also provide financial help, but it is very limited, because we want to make them independent. (G2P7)

Above, the social worker constructs her position by describing the circumstances pertaining to women’s limitations in striving for independence. The core of personal empowerment was around processes during which a woman started to take control over her own life and achieve independence from her husband. Leaving home was not a prerequisite for independence. Instead, participants highlighted independence in terms of earning money, getting an education, getting peer support from self-help groups, or accessing health care for a better quality of life. The social worker’s duties are described through moral positioning, as the social worker describes the duty to avoid creating a situation in which the survivor becomes too dependent on the social worker. This suggests that, occasionally, it might be appropriate for social workers to distance themselves from survivors to facilitate personal responsibility and empowerment, even if this distance might contradict the moral position and the duties of social workers to always be available to their clients. This demonstrates the social worker’s views on the duty to help survivors as well as the right to step back.

In contrast, workers also explained their duty to take a more active expert position. Helping a person make as informed decisions as possible introduces the possibility for participating in the decision-making process. The duty was not in giving solutions, but in clarifying the possible consequences and providing support.

They are not decision-makers. Since childhood, somebody else makes decisions. Very rarely do women make their own decisions. So, when you have to decide by yourself, it’s a big challenge for these women. But when she makes a decision and moves in that direction, the confidence that she gains is something that sustains her for life. (G2P8)

However, as stated above, the subservient role with which women have been brought up posed challenges when workers shifted their position from expert to facilitator, aiming to give women the space to make their own decisions. Traditionally, Indian women are not treated as independent subjects in their communities. Thus, a situation in which women should suddenly start acting as if they have power over their own lives can be confusing. Accordingly, as described below, empowering a survivor was a process by its very nature.

A client came to us on day one. Crying, crying, and crying. And then we can slowly, slowly see improvement, there is improvement in her self-esteem, confidence, that’s what we are talking about. That is something that then drives conclusions that [decisions] are made by them. Not by me. What I did was I facilitated the entire process. I let the client’s emotions flow—I didn’t stop her. (I3)

So there are cases of counseling, counseling skills, definitely we use that. Catharsis to emotional ventilation the client. Definitely we provide … that it is our first goal. To get catharsis. (G1P4)

In the first excerpt, the participant is referring to a previous episode, in which the social worker explicitly positions herself as someone who has succeeded in promoting personal empowerment by reflecting on her position in the process. Instead of merely describing the events and the position of the survivor, the position of the social worker in both extracts is reflected upon in relation to the moral order to support survivors and their personal empowerment. In this, enabling expressions of emotions aroused from the experiences of being exposed to violence is essential. This implies the social worker’s duty to help with the process, while having the right to step back and let the survivor take charge of her own life.

### Self-Reflector of Personal Emotions and Attitudes

In accordance with the story line of reflectivity, the theme of a social worker’s self-understanding helped to unfold participants’ attitudes and emotions regarding DV. In the participants’ explicit reflections, nonjudgmental attitudes were discussed. As can be seen below, this was highly valued, especially in situations in which a survivor was not yet able to leave her violent spouse.

When I sit there as a social worker, I have to believe her. Once I believe her, I trust her, that’s the most important thing. (I4)

I don’t think any one of our counselors asks why didn’t you step out earlier … or what did you do to aggravate him. They will simply never ask. (G2P2)

As a part of respecting the survivor’s autonomy, workers explicated the importance of avoiding requesting a survivor to leave a violent relationship against her own will and without in-depth consideration. A divorced woman living alone may be very vulnerable. By leaving home and living without her legal husband, a survivor may be at risk of being harassed. The high tolerance of survivors to violence was usually explained by the needs of their children. Staying in a violent relationship is seen as a better option than letting children grow up without a father. Besides, economic resources do not necessarily allow for new housing arrangements, which also affects the woman’s chances of getting custody. For example, only 61% of women in urban areas and 38.5% of women in rural areas had a bank account or savings of their own (IIPS & ICF, 2017).

Still, some participants expressed feelings of anger and frustration when a woman chose to go back to her violent partner. Nonetheless, these feelings did not necessarily indicate a victim-blaming mindset.

She said she had to go back because her children were there. So initially I was angry with her, but after that … power, that strength, that resilience … I realized, when working, that the entire belief that she’s a victim was totally shattered, because they are not victims, they are very strong people. I was very … inspired and shocked and surprised, because I used to think that they were weak … So, they don’t give up. Not even if the whole world is against them and everybody is blaming them, she is blaming herself but … she won’t give up. (I2)

Here, self-reflection on one’s own emotions also took place in the context of identifying professional development. A participant reflected on a process during which her capability to see beyond feelings of anger and frustration evolved. Instead of labeling a survivor as a helpless victim, the participant focused on the survivor’s strength and ability to cope with a very difficult situation. This involved ways of adjusting emotional responses according to expectations relating to professional practice and conduct as can be seen from discourse below.

We have to control our feelings. When we go home, we forget, leave things behinds us. (G2P7)

As professionals, we talk with them [emotions] but we don’t involve … (G2P5)

When you realized that you emotionally involved to your case, you kind of have to tell yourself it. It’s … It’s difficult … I think all the time we have to learn how to do it. See, we come up with the set of emotions, in any day, I mean there are issues that we are facing, in our personal lives also. So one way is that when you come here and start listen to other’s problem, you’ll forget your own problems, but chances that you’ll involve with your emotions and feelings of your clients are very high, especially in very sensitive cases. (G2P1)

Emotions arising from personal experiences of violence were expressed in discussions, even if they were not explored as separate questions by the interviewer. As can be seen below, earlier life experiences, whether emotions of exceptionally strong safety or ultimate fear, influenced the participants’ current practice.

For me, personally, violence was never a part of it, for generations. So … I know how happy my family is, how good a connection we have, that’s something I want to see. So, what I wish is that every woman was confident enough to not be dependent on somebody … So that’s what I hope my work means to somebody else. (G2P2)

So, whatever they told me, I had experience of my own. I was also a victim … I could feel it [pain] because I was one of them. But I didn’t have the privilege of sharing my story, because you have to be a professional. I just tried to do my work, and it has made me who I am today. (I3)

Feelings of safety and equality in childhood brought up a social worker’s personal work aims such as the drive to promote personally experienced independence. In contrast, one participant, as a survivor of long-lasting violence, based her strength as a professional on her process of overcoming the violence. The latter extract also demonstrates the juxtaposition between experience and professionalism. The worker had no right to express her personal experiences in client meetings. The possibility of sharing her experiences was referred to as a “privilege.” Instead, her duty to act professionally obliged her to put her own emotions aside. Earlier experiences of violence may have a negative effect on one’s professional work (Pecnik & Bezensek-Lalik, 2011). This highlights the reflection on one’s own emotions (O’Leary et al., 2013), which is not only a right but also a duty when working with survivors.

## The Limitations and Evaluations of Cross-Cultural Research

This was a small-scale study, and participants were recruited from NGOs located in an urban area in the state of Maharashtra. Individuals working in special DV units were presumed to have the best information on the topic, but the implications of the results for social work in the Indian public sector or in rural areas cannot be taken for granted. This research was conducted in a specific cultural context. However, universal themes—gendered power, women’s rights, women’s agency, and the worker’s self-understanding—are also applicable to practitioners in other cultural contexts as they make sense of DV and the rights and duties of social workers to intervene.

In terms of the trustworthiness of this study, issues regarding credibility, transferability, and dependability (e.g., Shenton, 2004) should be considered. The use of positioning theory as an established methodology, with the first author familiarizing herself with the culture, the triangulation of data, and the peer scrutiny of the three authors, aimed at achieving credibility for the article. Using a purposive sample might be considered a limitation in this regard, but it allowed the authors to focus on the specific context presented in the article from the viewpoint of the participants. Transferability was not aimed at in this article. Rather, the findings present a sample of cultural practices that adds to the understanding of the Indian context of DV work. Regarding dependability, we have reported the procedural steps of the study in as much detail as possible.

In addition, we made analytical generalizations of the data through category zooming and positioning (Halkier, 2011). Category zooming refers to the process of focusing the analysis on a specific theme of the study, in this case, the self-positioning of social workers. This was followed by analytical generalizations through positioning by analyzing the different forms of positioning through which the categories of self-positioning were constructed. This resulted in highlighting “the communicative dynamisms that are coconstitutive of social constructions of categories, relationships and performances” (Halkier, 2011, p. 793).

## Discussion and Implications for Practice

This study approached the question of how Indian social workers made sense of DV by investigating their self-positioning, including their emotions, attitudes, and practices in relation to their rights and duties. The findings show that professional self-positioning has potential implications for work with survivors. The self-positioning reflected political, cultural, and social themes, and the psychological dimensions in terms of the psychological impact of trauma were conspicuous by their absence. According to Tseris (2019), the concept of “trauma” ignores the actual violent act and the abuse itself, shifts the focus from gender-based issues to psychiatric ones, and thus limits the ability to refer to patriarchal structures as the basis of the problem. In line with the idea of other-positioning (Harré & Van Langenhove, 1999), self-positioning also implicates the position of the DV survivor. Thus, we can ask whether employees wanted to avoid labeling speech by not using the term “trauma” because of the stigma associated with mental health–related issues in India (Sayani, 2018).

However, as facilitators of personal empowerment, the position concerned the survivors’ personal growth and enhanced their agency by building their autonomy through self-determination and independent decision-making. Accordingly, elements of trauma-orientated work are apparent (Anyikwa, 2016). The aspect of post-traumatic growth was, however, more visible. As self-reflectors, participants emphasized the resilience of survivors, including their ability to cope with traumatic situations and how to turn personal experiences into resources, even in their own work (see also Pecnik & Bezensek-Lalik, 2011). Special training programs that social workers run for survivors who want to voluntary organize peer groups as grassroots help in communities offer women the opportunity to construct survivor identities as a part of their post-traumatic growth. In these activities, facilitating can be seen as parallel to and overlapping with advocating (e.g., Lapierre & Côté, 2011). Nevertheless, from the perspective of professional boundaries and client participation (O’Leary et al., 2013), social workers, as advocators of women’s rights, took an expert position as *representatives* of women. In advocating work with young boys and engaging male perpetrators to emancipation work with women, social workers encourage men to question traditions of masculinity/femininity to construct more egalitarian roles and responsibilities (see also Pandya, 2014).

As challengers of gendered oppression, social workers see beyond individual factors and recognize those structural issues such as patriarchy that are causes of violence. However, by applying the concept of forced self-positioning (Harré & Van Langenhove, 1999), we ask whether the patriarchal traditions not only deprive survivors of power, but also force social workers into a position in which they recognize male dominance; at the same time, their resources to act are limited. Accordingly, a survivor may be positioned paradoxically as an oppressed victim. This unintentional reproduction of boundaries, dividing dominant groups from the “other,” emphasizes the significance of reflexivity in terms of critically analyzing power relations and social divisions such as gender in direct practice (Mohanty, 1995; Pease, 2010). However, as challengers, none of the workers set leaving home as a prerequisite for help (compare with Keeling & Van Wormer, 2012). Participants were sensitive to the survivor’s decision to save her marriage. This located the woman’s individual experience such as her unwillingness to leave her husband in a wider theoretical context, highlighting male privilege and gendered divisions of power (Pease, 2010; Thompson, 2011). Thus, feelings of frustration and anger, expressed from the position of a self-reflector of personal emotions and attitudes, do not necessarily predict a victim-blaming mindset. Instead, in work with survivors, social workers should recognize that these feelings can be part of the process of respectful acceptance (Kulkarni et al., 2012). A worker can learn to see contextual issues behind the decision not to leave and to consider a survivor as an expert of her own situation by accepting that a woman has the right to make her own choices, even bad ones; bad choices do not justify a professional’s decision to withhold help (Lapierre & Côté, 2011).

The data generated from such a small-scale, qualitative study do not allow us to undertake investigation of the influence of gender and previous work experience on social workers’ rights and duties. However, the study does help to raise questions in relation to both points for further understanding of the social workers’ response in DV and the ways to develop existing service system.

To sum up, the results of this study reveal that practitioners encountering survivors can be in an important societal position, when it comes to raising awareness of human rights in DV cases. In line with Rajan (2018), the results highlight that dismissive attitudes toward women’s rights, underpinned by gendered stereotypes and roles, need to be challenged at their roots. Simultaneously, a study of this nature raises the question whether, in direct practice, we recognize that an uncritical application of Western principles when working with survivors may risk further “colonialization” if the expectations are culturally insensitive and based merely on an ideal of Western understanding (Mohanty, 1995; Rajan, 2018). Noting the sociocultural background of the survivor’s decisions (i.e., factors such as the stigma of divorce) does not exclude, but rather forces, a re-definition of concepts such as independence as a goal of the work. Instead of being related to the dilemma of staying or leaving, thus emphasizing the ideology of individualism from Western scholarship (e.g., Nikupeteri, 2017; Virkki & Jäppinen, 2017), independence as an ultimate goal was approached through the woman’s own will, and her discovery of it, by enhancing the woman’s social, psychological, and economic resources, and by creating possibilities to establish an independent space in which to live and make decisions (see also Pandya, 2014).

However, to improve practitioners’ opportunities to do their valuable work and exercise their professional positions, as presented in this article, importance of resourcing and supporting the implementation the Domestic Violence Act should is required. A wide range of state measures, such as social protection policies and programs for the vulnerable groups exposed to violence, should be enhanced. As a part of this initiative, the role of NGOs as key services providers should be acknowledged and resourced and collaboration and partnership between government and NGO sectors enhanced. To support United Nations (2010) aims, regular professional trainings targeting DV authorities and services should be rolled out nationally. In addition, more comprehensive collection of comprehensive statistics and data is required for evaluation, monitoring, and planning purposes.
